# Unilateral Magnetic Resonance‐Guided Focused Ultrasound (MRgFUS) Pallidothalamic Tractotomy in Parkinson's Disease: Promising Results But Uncertain Target

**DOI:** 10.1002/mds.70131

**Published:** 2025-11-18

**Authors:** Mickael Aubignat

**Affiliations:** ^1^ Department of Neurology Amiens Picardie University Hospital Amiens France; ^2^ Research Unit UR‐4559 (LNFP) Laboratoire de Neurosciences Fonctionnelles et Pathologies University of Picardie Jules Verne Amiens France

I read with interest the recent article by Ikezawa et al. on unilateral magnetic resonance‐guided focused ultrasound (MRgFUS) pallidothalamic tractotomy for Parkinson's disease.^1^ The authors demonstrated meaningful motor improvements (including bilateral benefits) after unilateral lesion in Forel's field H1. I wish however, to raise a constructive caution regarding the interpretation of these results. In particular, the lack of detailed lesion verification (notably the absence of diffusion tensor imaging to confirm tract‐specific targeting) leaves uncertainty as to whether the clinical effects can be attributed to selective disruption of the intended pathway alone. Without advanced neuroimaging validation, one must be careful in drawing mechanistic conclusions about ‘pallidothalamic tractotomy’ in isolation.

The subthalamic region targeted in this procedure is an anatomically complex and crowded crossroads of structures and fiber pathways.^2^, ^3^, ^4^, ^5^ The classic fields of Forel in the posterior subthalamic area form a narrow ‘bottleneck’ through which multiple myelinated tracts converge to the thalamus. These include the pallidothalamic fibers (ansa lenticularis and lenticular fasciculus merging into Forel's field H1), but also the cerebellothalamic tract running just posterior to the pallidal fibers, as well as nigrothalamic projections from the substantia nigra coursing in parallel. In addition, key gray matter nuclei (zona incerta [ZI] and subthalamic nucleus) lie immediately adjacent to these tracts. Notably, the ZI lies between the lenticular and thalamic fasciculi, in extremely close proximity to the pallidothalamic bundle. Recent anatomical and surgical reviews of the posterior subthalamic area underscore this intricate anatomy and the challenge of achieving a truly selective lesion highlighting the potential for off‐target effects if a lesion spreads beyond the intended bundle.^2^ Likewise, Horisawa et al. noted that ablating the pallidothalamic zone in this region often inevitably involves or affects the ZI, making it “difficult to distinguish” which structure's disruption is actually responsible for clinical benefits.^5^ This issue is highly pertinent to interpreting the results of Ikezawa et al., since without tract‐specific imaging we cannot be certain whether their MRgFUS lesion was confined to the pallidothalamic tract as presumed, or whether it also partially lesioned adjacent fiber tracts or nearby nuclei.^1^ In short, the subthalamic target area's complexity demands modern imaging confirmation to validate that the ‘pallidothalamic tractotomy’ is anatomically what its name implies.

The authors reasonably hypothesize that interrupting pallidal outflow fibers led to the beneficial outcomes. However, without supportive neuroimaging evidence this remains an inference. For example, one could argue that a lesion extending slightly posteriorly into the cerebellothalamic tract might also reduce tremor and rigidity, or that involvement of the ZI could contribute to improvements in axial symptoms. Indeed, contemporary imaging studies have shown that the pallidothalamic and cerebellothalamic tracts run only millimeters apart and even small lesions might affect both if not precisely placed.^2^ In my view, the impressive clinical outcomes reported would be more convincing as evidence of a specific pallidothalamic mechanism if corroborated by tract‐based imaging confirmation.

## Author Roles

(1) Research Project: A. Conception, B. Organization, C. Execution; (2) Statistical Analysis: A. Design, B. Execution, C. Review and Critique; (3) Manuscript Preparation: A. Writing of the First Draft, B. Review and Critique.

M.A.: 1A, 1B, 1C, 3A.

## Full Financial Disclosures for the Preceding 12 Months

The author declares that there are no additional disclosures to report.

## Data Availability

Data sharing not applicable to this article as no datasets were generated or analysed during the current study.
